# Nanoapplication of a Resistance Inducer to Reduce *Phytophthora* Disease in Pineapple (*Ananas comosus* L.)

**DOI:** 10.3389/fpls.2019.01238

**Published:** 2019-10-11

**Authors:** Xinhua Lu, Dequan Sun, James E. Rookes, Lingxue Kong, Xiumei Zhang, David M. Cahill

**Affiliations:** ^1^Key Laboratory of Tropical Fruit Biology, Ministry of Agriculture, South Subtropical Crop Research Institute, Chinese Academy of Tropical Agricultural Science, Zhanjiang, China; ^2^School of Life and Environmental Sciences, Deakin University, Geelong, VIC, Australia; ^3^Institute for Frontier Materials, Deakin University, Geelong, VIC, Australia

**Keywords:** mesoporous silica nanoparticles, resistance inducers, salicylic acid, gatekeeper, *Phytophthora*, pineapple

## Abstract

Treatment of plants with a variety of abiotic and biotic inducers causes induced resistance to pathogen attack. In this study, the effect of four resistance inducers on plant diseases caused by *Phytophthora cinnamomi* was screened *in vivo* initially by using lupin, a susceptible model plant. Lupin pretreated with 0.5 mM salicylic acid (SA) showed effective resistance against *P. cinnamomi* with restricted lesions. Then, mesoporous silica nanoparticles (MSNs) with particle size around 20 nm and approximate pore size of 3.0 nm were synthesized and functionalized for loading and importing SA to pineapple plantlets. Decanethiol gatekeepers were introduced to the surface of MSNs *via* glutathione (GSH)–cleavable disulfide linkages to cover the pore entrance, which was confirmed through using Raman spectroscopy. Through free diffusion, the loading efficiency of SA in MSNs gated with gatekeepers was 11.7%, but was lower in MSNs without gatekeepers (8.0%). In addition, *in vitro* release profile of SA from gatekeeper-capped MSNs indicated that higher concentrations of GSH resulted in more cargo release. Moreover, the experiments *in planta* showed that the application of MSNs as a resistance inducer delivery system significantly improved pineapple resistance to *P. cinnamomi* in terms of inhibiting lesion development and improving root growth of infected plants, compared to the use of free SA and MSNs without gatekeepers. The analysis of SA, GSH, and defense-related genes, of *PR1* and *PR5*, further confirmed that the slow and prolonged release of SA from MSNs inside the roots of pineapple plants was achieved through a redox-stimuli release mechanism. Therefore, the application of MSNs with redox-responsive gatekeepers has shown great potential as an efficient tool for delivering chemicals into plants in a controllable way.

## Introduction

*Phytophthora cinnamomi*, a plant pathogen with a worldwide distribution, infects thousands of plant species, including trees, woody shrubs, and herbs (Cahill et al., 2008; Allardyce et al., 2012). *Phytophthora cinnamomi* invades roots, grows inside the root system, and causes root rot, which impedes water uptake and transport to the shoot, finally leading to wilting and chlorosis of the foliage (Hardham and Blackman, 2018). The long-term survival ability of the pathogen is attributed to the fact that it can grow symptomlessly in infected plants or in the soil, which makes the complete eradication of the disease extremely difficult (Jung et al., 2013). Currently, *P. cinnamomi* causes destructive plant disease in areas with a Mediterranean climate, including parts of the United States, Australia, Mexico, and the Iberian Peninsula (Burgess et al., 2017). The pathogen not only causes considerable economic losses in agriculture, horticulture, and forestry, but also leads to disastrous consequences for biodiversity and natural ecosystems.

Pineapple (*Ananas comosus* L.) is the third most important fruit crop in the tropical and subtropical regions of the world, only preceded by banana and citrus (Lu et al., 2014). However, heart and root rot disease of pineapple caused by *P. cinnamomi* results in serious losses and greatly limits the development of the pineapple industry in a number of countries (Anderson et al., 2012). Compared to other crops, pineapple leaf and canopy transpiration rates are very low. The accumulation of water in the top soil is common, especially when the plantlets are planted at high density, leading to a conducive environment for *Phytophthora* infections. Moreover, new roots of pineapple are highly susceptible to root pathogens such as *P. cinnamomi* (Rohrbach and Johnson, 2003). Currently, there is no effective method available to eradicate *P. cinnamomi* without destroying the infected plants. Most of the current control measures aim to reduce the spread of the pathogen and to improve plant defense *via* cultural or chemical methods (Gunning et al., 2013). Approaches to alleviate the damage caused by *P. cinnamomi* in pineapple include growing the plants in aerated, raised and well-drained beds and supplementing soil with elemental sulfur, which reduces the soil pH below 3.8. However, sulfur may cause adverse effects on plant nutrition as the low pH value inhibits the absorption of important nutrient elements such as potassium, magnesium, and calcium (Anderson et al., 2012).

Studies have shown that the exogenous application of a number of compounds that act as resistance inducers or “primers,” may induce plant defense including broad-spectrum systemic acquired resistance (SAR) and/or induced systemic resistance to pathogen infection (Walters et al., 2013). However, the effects of these resistance inducers are greatly limited when conventionally used because a large amount of the chemical cannot be introduced into crops due to the low uptake rate and high cost. Moreover, resistance inducers tend to be decomposed or removed by both biotic and abiotic factors before they are absorbed into crops (Mukhopadhyay, 2014). Recently, the rapid development of nanotechnology has offered new methods to address these issues. Among all the engineered nanoparticles, mesoporous silica nanoparticles (MSNs) are especially attractive as a novel biomolecule carrier because of their unique properties. The application of MSNs as a controlled drug delivery system to import anticancer drugs into various mammalian cells and tissues has been widely reported (Li et al., 2016; Freitas et al., 2017; Khosravian et al., 2018). However, the utilization of MSNs to transport agrochemicals is still at the initial stage. In one study, MSNs were applied for the encapsulation of 2,4-D sodium salt. The controlled release of the herbicide was achieved, and the MSN-based system greatly reduced the leaching from soil of the entrapped chemical (Cao et al., 2017). Recently, MSNs were employed to transport the phytohormone abscisic acid (ABA), and the release of the imported ABA was demonstrated in the model plant *Arabidopsis thaliana* (Sun et al., 2018). However, until now the application of MSNs as a controlled delivery vehicle for importing resistance inducers to control plant disease has not been reported.

In this study, four resistance inducers, salicylic acid (SA), methyl jasmonate (MeJA), phosphite (Phi), and dl-β-aminobutyric acid (BABA), were screened in lupin plants following inoculation with *P. cinnamomi*. Monodispersed MSNs with a particle and pore size of approximately 20 and 3.0 nm, respectively, were synthesized and functionalized. To achieve the aim of controlled release of the entrapped cargoes, the concept of gatekeepers was applied. Decanethiol gatekeepers were attached to the surface of MSNs *via* GSH-cleavable disulfide linkages. The loading efficiency (LE) of SA and *in vitro* release pattern in the presence of different levels of glutathione (GSH) were determined. Finally, SA was imported into pineapple plantlets using a MSN-mediated delivery system with redox-responsive gatekeepers. The efficiency of this novel delivery system for the control of plant disease caused by *P. cinnamomi* was evaluated through the analysis of different parameters related to infected root growth and development. In addition, the contents of SA and GSH in the tested plants were measured. The expression of pathogen defense-related genes *PR1* and *PR5* in the treated roots was also examined and quantified. We herein report that the MSN-mediated delivery system with redox-responsive gatekeepers greatly enhances the ability of resistance inducers to control plant disease. Hence, the study demonstrates the potential application of MSNs in agriculture.

## Materials and Methods

### Growth and Maintenance of Experimental Plants

Lupin [*Lupinus angustifolius* var. Wonga] seeds were purchased from Greenpatch Organic Seeds (Taree, New South Wales, Australia). First, lupin seeds were submerged in distilled water for 5 h and then sterilized for 2 min with sodium hypochlorite (1%, v/v), followed by rinsing with tap water for 5 min to remove any residual disinfectant. Next, the seeds were placed in a tray on a multilayer of paper towel, which was moistened with distilled water. The tray was then covered with aluminum foil and incubated at 22°C in the dark. After 2 days, germinated seeds with a uniform radical length of about 0.5 cm were selected for placing in a soil-free plant growth system according to Gunning and Cahill (2009). Nutrient solution (Total Horticultural Concentrate; Excel Distributors, Reservoir, Australia) was applied once a week following the manufacturer’s recommendations. Lupin plantlets were transferred into a growth cabinet (Thermoline, Coburg North, Australia) at 22°C with 16/8-h photoperiod with light intensity of 250 to 300 μmol m^−2^ s^−1^.

Plantlets of pineapple (*A. comosus* cv. “MD-2”) were propagated using a leaf-bud cutting method (Seow and Wee, 1970). Pineapple crowns were kindly provided by Mr. Col Scott (Tropical Pines Pty Ltd., Queensland, Australia). In general, an active bud may grow into a pineapple plantlet because the photosynthesizing leaf attached to the bud provides the bud with nutrition. The pineapple propagation method using leaf buds is presented in **Supplementary Figure S1**. A single leaf with an axillary bud (leaf bud) at the base was carefully dissected from the pineapple crowns. The leaf-bud cuttings were submerged in a 0.1% potassium permanganate solution for 1 h. Then, the cuttings were rinsed with tap water for at least 5 min and allowed to air dry. The cuttings were then planted in pots with sterilized medium (sand:peat 50:50). They were kept in the growth cabinet, maintaining temperature at 25°C with 16/8-h photoperiod at 250 to 300 μmol m^−2^ s^−1^. Water was sprayed every other day, and 500 ml water was added into the container twice each week to keep the growth medium wet. In addition, nutrient solution was applied once a week. The buds developed into plantlets in 2 to 3 months post planting (MPP). After the plantlets grew to about 6-cm height, they were carefully detached from the joined leaves and transplanted into soil (Debco® Premium potting mix; Debco, Victoria, Australia), with one plant per pot (6-cm diameter) and then were returned to the above growth conditions. Water and nutrient solution were supplied regularly. At 3.5 to 4 MPP, the plantlets about 10 cm in length were removed from soil and rinsed with tap water. All the primary roots were cut off. Finally, the plantlets without roots were transferred into the soil-free plant growth system to develop new clean roots for pathogen inoculation.

### *P. cinnamomi* Preparation

The *P. cinnamomi* A2 isolate DU046 was from the Deakin University culture collection. The isolate was maintained on 10% clarified V8-juice (CV) agar medium at 24°C in the dark and subcultured every 7 days. Zoospores of *P. cinnamomi* were obtained according to the method of Byrt and Grant (1979). The density of zoospores was determined using a hemocytometer, and inoculations were performed at 1 × 10^5^ spores/ml.

### Resistance Inducer Screening on *P. cinnamomi* Infected Lupin Roots

The activities of resistance inducers were investigated by using lupin, a *P. cinnamomi*-susceptible model species. The resistance inducers tested in the study were SA, MeJA, Phi, and BABA. In this study, “Throw Down” systemic fungicide was purchased as the source of Phi (YaraNipro Pty Ltd, Queensland, Australia), with the active content of 400 g/L phosphorous acid (H_3_PO_3_), present as mono (KH_2_PO_3_) and dipotassium phosphite (K_2_HPO_3_). Different concentrations of SA (0.1, 0.5, 1.0, 5 mM), MeJA (0.1, 1.0, 10, 100 µM), Phi (0.01, 0.1, 1, 10 g/L), and BABA (0.01, 0.1, 1, 10 g/L) were prepared using sterile distilled water (sdH_2_O). For SA and MeJA, they were dissolved in a small volume of absolute ethanol first and then sdH_2_O was added to the desired concentration.

Five-day-old lupin plantlets were sprayed with the four inducers using a hand spray bottle (10 ml), with three sprays for both sides of each leaf. As a control, sdH_2_O was directly sprayed to the plants. After 2 days, each root was inoculated with a 20 µl drop of *P. cinnamomi* zoospore suspension (1 × 10^5^ zoospores/ml), placed 5 mm behind the root tip. Each experiment included three biological replicates. A replicate consisted of 12 lupin plantlets. Untreated plants were included as a control treatment. At 6 days post inoculation (dpi), root growth and lesion length were recorded, along with the incidence of disease and the number of lateral roots formed. Disease severity was graded from 0 to 5 according to lesion length: 0, no visible symptoms; 1, mild, lesion length on <20% of the root length; 2, mild to moderate, lesion length on 20-30% of the root length; 3, moderate, lesion length on 30% to 40% of the root length; 4, moderate to severe, lesion length on 40% to 50% of the root length; 5, severe, lesion length on >50% of the root length.

### Synthesis of MSNs as a Controlled Agrochemical Delivery System

The synthesis and modification of MSNs before loading resistance inducers were conducted according to the previous methods (Yi et al., 2015; Sun et al., 2018). To link redox-responsive short-chain gatekeepers, thiol groups were first introduced at the pore entrance of the particles, which contained the template of cetyltrimethylammonium bromide (CTAB) inside. Typically, 200 mg of MSNs were suspended in 200 ml of ethanol, followed by adding 0.2 ml of (3-mercaptopropyl) trimethoxysilane (MPTMS) into the mixture dropwise under stirring. After 1 h, 2 ml of Milli-Q water was added, and the resulting solution was stirred for 24 h at room temperature. Subsequently, the solution was heated to 80°C to stabilize the silanol groups between MSNs and MPTMS. The resulting powder was collected by centrifugation at 8000*g* for 10 min and then was washed with ethanol. The as-prepared particles were resuspended in a solution containing 100 ml of ethanol and 1 ml of hydrochloric acid (32%), under vigorous stirring and heated at 60°C for 12 h. The final thiol group functionalized MSNs without CTAB template were obtained after centrifugation, washing, and drying. These MSNs were designated as MSN + SH.

According to resistance inducer screening, SA was employed as a loaded and imported cargo. The cargo loading procedure was performed through a free diffusion method. Briefly, 200 mg of SA was dissolved in 10 ml of absolute ethyl acetate (EA). Then, 200 mg of MSN + SH was added to the SA-EA mixture solution. The mixture was sonicated for 30 min and kept in a rotating shaker at room temperature for 24 h, so that the SA molecules entered into the pores completely through free diffusion. A blank containing MSN + SH in EA was also prepared. Afterward, 0.5 ml of didecyl disulfide was added into the above mixture to react with the thiol groups to produce the “disulfide bridge” gatekeepers. The reaction was continued at the same condition for another 24 h. Then, the excessive SA and EA were removed by centrifugation and repeated washing with 40% (v/v) ethanol. Subsequently, the resulting particles were quickly frozen in liquid nitrogen to keep the cargo inside the mesopores and then dried in a freeze dryer for 36 h. The SA-loaded MSNs with gatekeepers were designated as MSN + SA + G. As a control, MSNs without gatekeepers were used to load SA, and the sample was designated as MSN + SA.

### Characterization

The morphology of MSNs after modification was observed by transmission electron microscopy (JEM-2100 electron microscope; JEOL, Japan) with an accelerating voltage of 200 kV. Surface changes and porosity of the NPs were analyzed by nitrogen adsorption–desorption isotherms analysis (Micromeritics Tristar 3000 Analyzer, Particle and Surface Science, UK) at −196°C under a static adsorption condition. The surface area and pore volume were calculated by the Brunauer–Emmett–Teller and Barrett–Joyner–Halenda (BET/BJH) analysis (Brunauer et al., 1938; Barrett et al., 1951), and the pore size distributions were obtained by the BJH method. Raman spectra were checked by using Raman microscopy (Renishaw, United Kingdom) with laser wavelength of 514 nm, equipped with a confocal microscope.

### MSN Molecular Loading and *In Vitro* Release

To determine the LE and loading capacity (LC) of SA, the amount of SA in the supernatant during the loading procedure was measured with a UV-visible spectrophotometer (Cary 300; Agilent Technologies, California, USA) at a wavelength of 305 nm. The LE and LC of SA in the NPs were calculated as follows: LE (%) = (weight of SA loaded in NPs/total amount of SA added) × 100%, LC (µg/mg) = weight of SA loaded in NPs/SA-loaded NPs.

The resistance inducer *in vitro* release was determined using a dialysis method. First, bare MSNs or gatekeeper-capped MSNs with SA (3 mg) were placed in a dialysis tubing (Sigma; MWCO 2,000 Da; St. Louis, Missouri, USA), which was wrapped by a steel mesh to hold it on the top of a cuvette. Then, the wrapped NPs were immersed in 3.5 ml water or different concentrations of GSH (1, 5, and 10 mM). At predetermined time intervals, 0.2 ml of the solution was collected in order to measure the released SA and then the fresh corresponding release solution was immediately replenished to maintain the same total solution volume. Salicylic acid in the solution was quantified to monitor the release. The cumulative molecule release was calculated according to the following equation:

$$\text{Amount of SA released (}\mu \text{g)}=\text{Vs}\sum_{\text{i}=0}^{\text{n-}1}\text{Ci}+{\text{V}}_{\text{0}}\text{Ci}$$

where *V*_s_ is the sampled volume collected at a predetermined time interval (*V*_s_ = 0.2 ml); *C*i (µg/ml) is the SA concentration released in the solution at time *i*; *V*_0_ is the volume of release solution (*V*_0_ = 3.5 ml); and *n* is the number of samples. For each resistance inducer, the measurements were performed on three replicates.

### Exposure of Pineapple to SA, MSNs, and MSN + SA + G

Prior to delivery to plants, SA was dissolved in a small volume of absolute ethanol, and then sdH_2_O was added to obtain the desired concentration. Mesoporous silica nanoparticles and MSN + SA + G were dispersed in sdH_2_O and sonicated for at least 2 h to form a homogeneous suspension. Based on previous experiments, the abaxial and adaxial leaf surfaces of pineapple plantlets were sprayed until runoff with free SA (0.5 mM), MSNs (0.5 mg/ml), or MSN + SA + G (0.5 mg/ml) containing 0.5 mM SA, respectively, using a hand spray bottle. Control leaf samples were treated with an equivalent amount of sdH_2_O. After being sprayed, plantlets were allowed to dry and then returned into the growth cabinet. After 3 days, root tips of the pineapple plantlets were inoculated with a 20 µl drop of *P. cinnamomi* zoospore suspension (1 × 10^5^ spores/ml) or water (mock inoculation). The experimental design and pineapple plantlets were treated as follows: noninoculated control, water-treated/mock inoculation (W–P); inoculated control, water-treated/*P. cinnamomi*–inoculated (W + P); SA-treated/mock inoculation (SA–P); SA-treated/*P. cinnamomi*–inoculated (SA + P); bare MSN-treated/mock inoculation (MSNs–P); bare MSN-treated/*P. cinnamomi*–inoculated (MSNs + P); MSN + SA + G–treated/mock inoculation (MSN + SA + G–P); and MSN + SA + G–treated/*P. cinnamomi*–inoculated (MSN + SA + G + P). Each experiment included three biological replicates. A replicate consisted of 10 pineapple plantlets, and two to three roots were treated per plantlet. Root samples were collected at the designated time point for direct use or frozen immediately in liquid nitrogen and stored at −80°C for further analysis.

### Assessment of Root Growth and Lesion Development in Inoculated Pineapple Plants

After incubation, the plants were checked for root growth and visible lesions every 2 days until 6 dpi. Percentages of lesion length to root length in controls and pathogen-inoculated plants were assessed, along with quantification of lateral root formation.

### Quantification of SA and GSH in Pineapple Roots Inoculated With *P. cinnamomi*

The content of SA transported into roots was determined using a high-performance liquid chromatography (Agilent Technologies) according to Pan et al. (2010) with minor modification. Briefly, approximately 100 mg of roots from each of three plants per treatment was frozen in liquid nitrogen in a 1.7 ml Eppendorf tube and ground to a fine powder. One milliliter of extraction buffer (2-propanol/H_2_O/concentrated HCl [2/1/0.002, v/v/v]) was added. The mixture was shaken at a speed of 100 rpm for 30 min. Then, 2 ml of dichloromethane was added into the mixture and incubated for another 30 m in the shaker at a speed of 100 rpm. Afterward, samples were centrifuged at 13,000*g* for 10 min, and 900 µl of the cleared liquid from the lower phase was carefully removed and placed into a fresh tube. The liquid was then air dried using a nitrogen evaporator. Finally, the samples were redissolved in 0.1 ml of 20% methanol and filtered through a 0.45-µm syringe filter and stored at −80°C until analysis. Fifty microliters of each sample was injected into the column for analysis. The concentration of SA was determined according to a standard curve derived from SA calibration standards.

In plant cells, GSH exists in reduced and oxidized forms. In this study, the total GSH (GSH + GSSG) in pineapple roots was used to assess the oxidative stress after pathogen inoculation. The GSH content was determined using a Glutathione Assay kit (Sigma).The sample was first deproteinized with a 5% 5-sulfosalicylic acid solution. Glutathione content of the sample was then assayed using a kinetic assay in which catalytic amounts of GSH cause a continuous reduction of 5,5-dithiobis-(2-nitrobenzoic) acid (DTNB) to TNB. In brief, roots (100 mg) from each of three plants per treatment were frozen in liquid nitrogen in a 1.7 ml Eppendorf tube and then ground to a fine powder. One milliliter of chilled 6% metaphosphoric acid was added, the suspension vortexed vigorously and then centrifuged at 14,000*g* for 20 min at 4°C. The supernatant was transferred into a fresh tube and kept on ice in the dark. The total GSH in the supernatant was quantified according to the manufacturer’s protocols. The fluorescent product was measured at 412 nm using a microplate reader (Thermo Scientific Variokan; Thermo Fisher Scientific, USA), and GSH content was determined using a GSH standard curve.

### Resistance-Related Gene Expression Analysis in Pineapple Roots Inoculated With *P. cinnamomi*

Resistance-related gene expression was analyzed by semiquantitative reverse transcription–polymerase chain reaction (PCR) in pineapple roots inoculated with *P. cinnamomi*. In brief, total RNA was extracted from 100 mg of pineapple root samples using Trizol (TRI Reagent®, Sigma). For cDNA synthesis, a Tetro cDNA synthesis kit (Bioline, Alexandria, New South Wales, Australia) was used according to manufacturer’s instructions. DNA sequences of pathogenesis-related genes *PR1* and *PR5* in pineapple were kindly provided by Dr. Lubing Zhang from Chinese Academy of Tropical Agricultural Sciences (China). Primers were designed using Primer3 version 0.4.0 and are listed in **Table 1**. Reverse transcription–PCR was conducted to evaluate the relative expression of *PR1* and *PR5* with Go Taq® Green Master Mix (Promega, Madison, WI, USA). Briefly, PCR reactions contained 10 μl GoTaq, 8 μl DEPC-treated water, 0.5 μl F primer, 0.5 μl R Primer, and 1 μl cDNA. Polymerase chain reaction conditions consisted of an initial denaturing step of 3 min at 95°C, followed by 31 cycles for *PR1*, 36 cycles for *PR5*, and 35 cycles for *actin*, at 95°C for 30 s, 62°C for 30 s, and 72°C for 30 s. The resulting PCR was run on a 1.8% agarose gel stained with EtBr and visualized under UV. Gene band intensity in the gels was measured by using ImageJ software, and quantification for relative gene expression was estimated against the reference gene (*actin*).

**Table 1:** Primers for genes used in the study.

Primer name	Oligonucleotide sequence (5′-3′)
*PR1*	F: TGCTATGCTTTGTGCCGTGT
	R: GATGTTCCCCGGAGGGTTAT
*PR5*	F: GTGAAATTGGTGCCGTCGTA
	R: GCCAGTCCTGGACCTTCAAC
*Actin*	F: CACTGTG CAATCTACGAGGGT
	R: CACAAACGAGGGCTGGAACAAG

### Statistical Analysis

All experiments in this study were conducted with three biological replicates. Data are presented as mean ± standard deviation. Statistical analysis was performed on the differences between the controls (CK) and treatments by using Duncan's multiple-range test at *p* < 0.05 (one asterisk) with IBM SPSS 24.0 software.

## Results

### Morphology and Physicochemical Properties of MSNs

**Figure 1:**
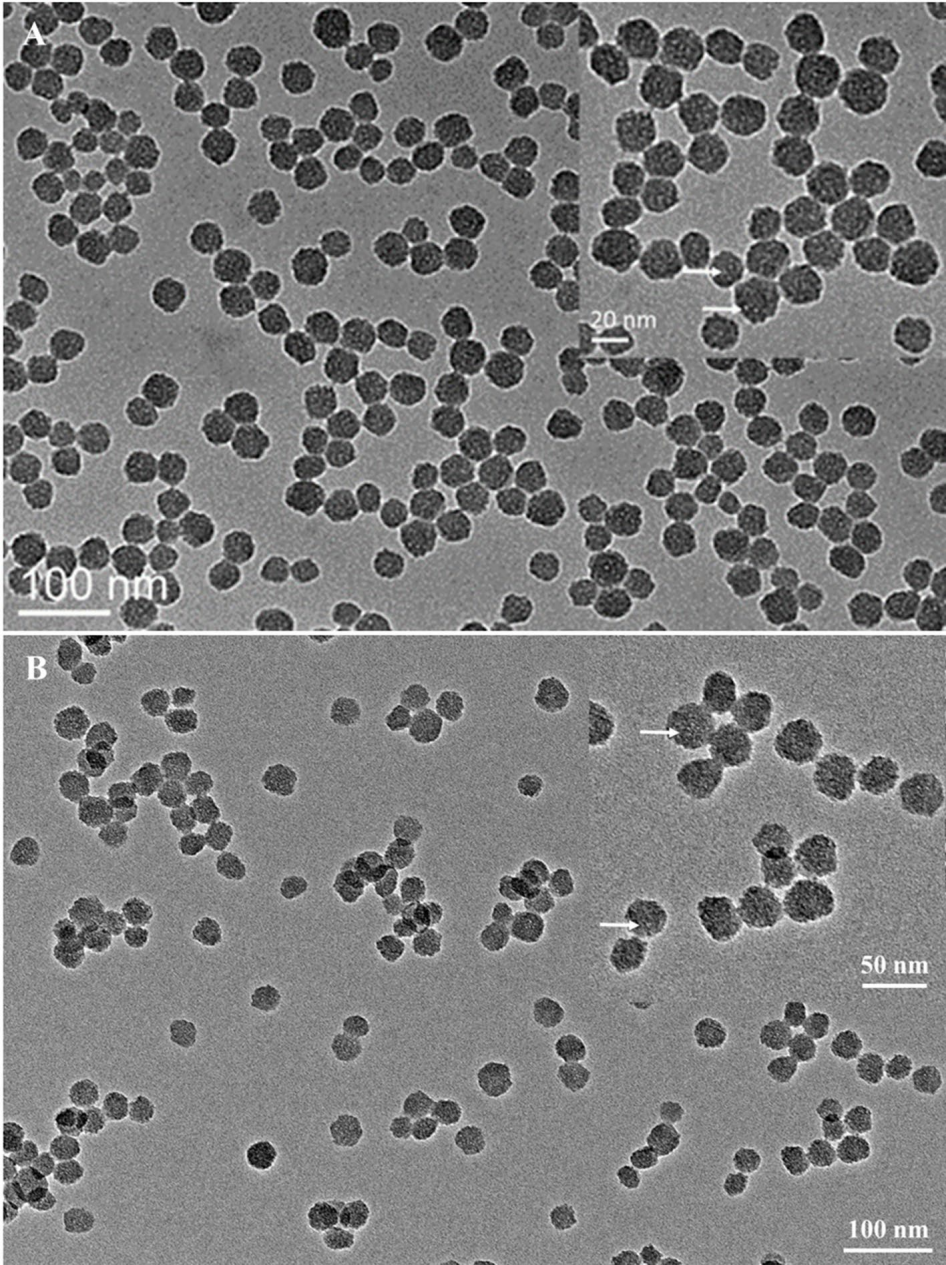
Transmission electron microscopy images of bare MSNs **(A)** and MSN + SH **(B)**. The inset figure shows the particles had a porous structure under high magnification (white dots indicated by the white arrows).

**Figure 2:**
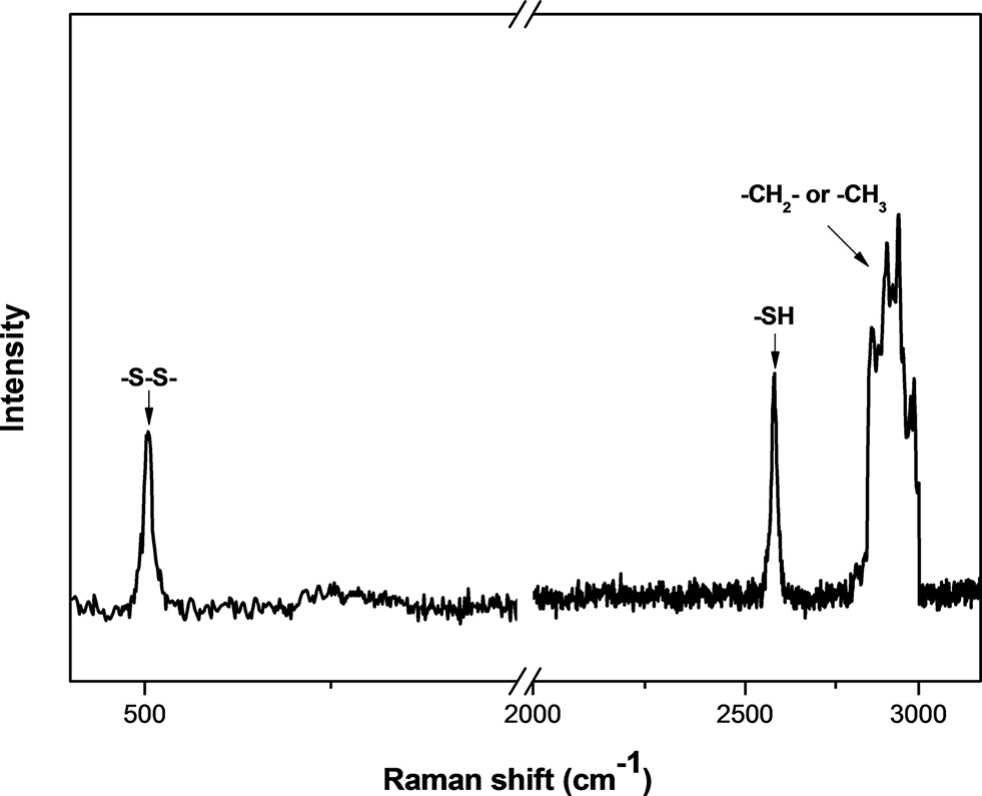
Raman spectra of gatekeeper-capped MSNs (MSN + G).

The size and shape of MSNs did not change significantly after being functionalized with thiol groups (**Figure 1**), and the mesoporous structure inside the MSNs could be clearly observed from the image. The particle mesoporosity was also confirmed by nitrogen sorption isotherms (**Supplementary Figure S2**) and BET/BJH method (**Supplementary Table S1**). The Raman spectra showed the characteristics of MSNs capped with gatekeepers (**Figure 2**). The absorption peak for disulfide bonds was observed between 500 and 550 cm^−1^ in the spectrum. After capping the gatekeepers, the peak around 2,600 cm^−1^ was still present in MSNs, which corresponded to the thiol groups of didecyl disulfide. In addition, the increased intensity of -CH_2_-/-CH_3_ peaks between 2,800 and 3,000 cm^−1^ could be attributed to introduction of the alkyl chain from decanethiol.

### Screening Resistance Inducers on Lupin Inoculated With *P. cinnamomi*

As shown in **Supplementary Figure S3**, no symptoms were observed for noninoculated control lupin. Inoculated control roots exhibited the most serious disease symptoms, which were present as water-soaked, discolored, long spreading lesions. Among the four resistance inducers (**Supplementary Figure S3** and **Table S2**), SA proved to be the most effective against *P. cinnamomi* and promoted root growth at the concentration of 0.5 and 1.0 mM after inoculation, showing restricted infection with more lateral root formation.

### SA Loading and Release

In this study, the LE of SA was 8.0% and 11.7% for the bare MSNs and gatekeeper-capped MSNs, respectively (**Supplementary Table S3**). So, approximately 88.8 and 134.1 µg SA/mg were loaded into MSN + SA and MSN + SA + G, which suggested that gatekeepers could block the pore entrance and prevent guest molecules from premature release.

**Figure 3:**
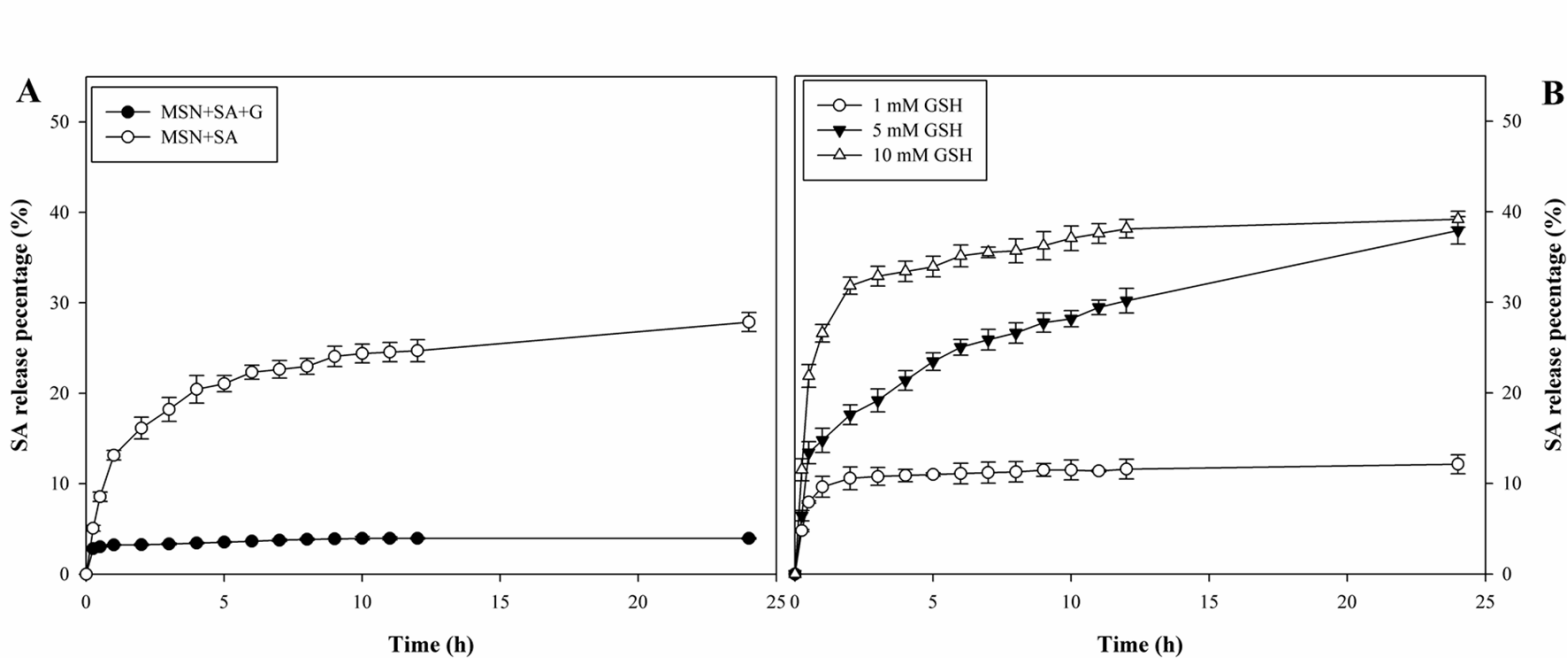
Redox-responsive release profiles of a resistance inducer from MSN-based vehicles. **(A)** Cumulative release of SA from MSN + SA + G with or without gatekeepers in the absence of GSH. **(B)** Cumulative release of SA from MSNs in GSH concentrations of 1, 5, and 10 mM. Data represent mean ± SD of three biological replicates.

In the absence of GSH, SA was released quickly from MSN + SA and reached 27.9% at 24 h (**Figure 3A**). In contrast, MSN + SA + G released only a small amount of SA at the beginning and then reaching a maximum value at 10 h (4.0%). However, as shown in **Figure 3B**, the leakage of SA from gatekeeper-capped MSNs exhibited a fast “initial burst” leakage in the first 1 h (14.8% and 26.6%, respectively) in the presence of a high concentration of GSH (5, 10 mM), and this was followed by a continuous release until 24 h (37.9% and 39.2%, respectively). In comparison, the SA release was characterized by a similar burst release in the first 1 h (9.6%) but was relatively slow at a low GSH concentration (1 mM).

### Symptom Development in *P. cinnamomi*–Inoculated Pineapple Roots Pre-Exposed to SA, MSNs, or MSN + SA + G

**Figure 4:**
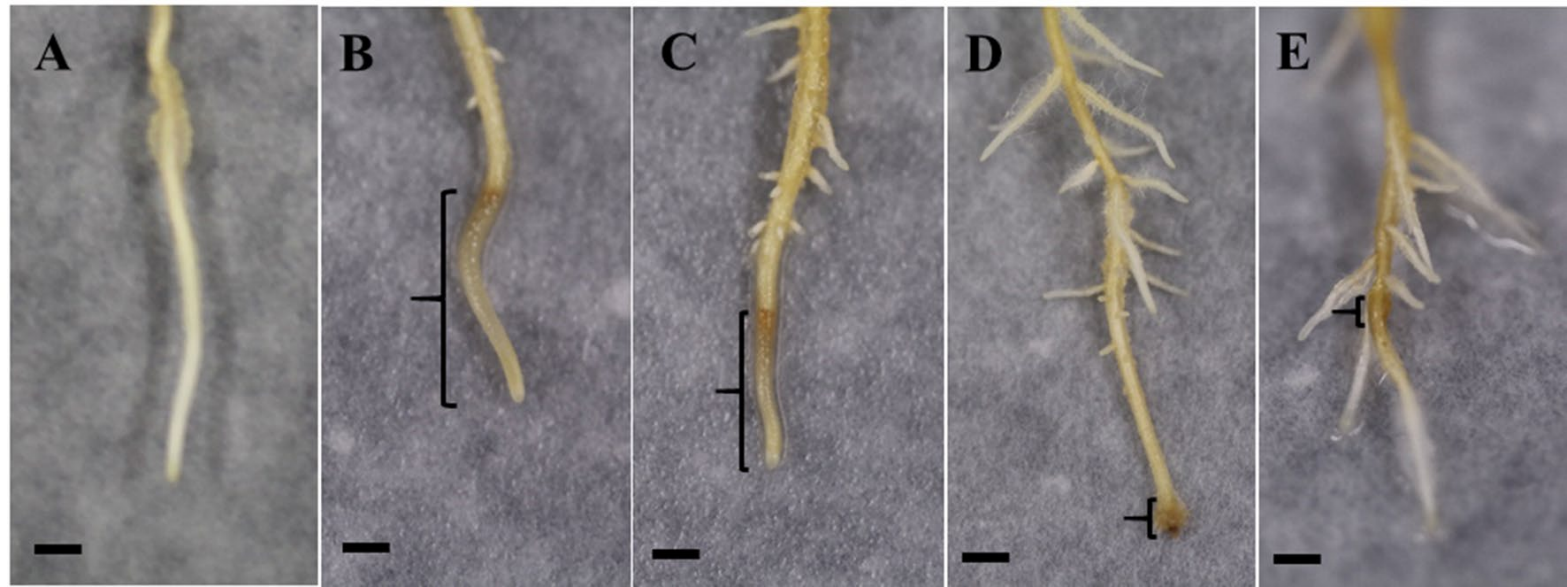
Representative images at 6 dpi of lesion formation in pineapple roots grown in the soil-free plant growth system and pre-treated with SA, MSNs, or MSN + SA + G, followed by inoculation with *P. cinnamomi* (P) zoospores (lesion is highlighted by the brackets). All treatments showed lateral root formation above the inoculation site. **(A)** No symptom in W–P. **(B)** Water-soaked, brown, spreading, long lesions in W + P. **(C)** Water-soaked spreading, short lesions in roots treated by SA + P. **(D)** Restricted lesion in roots treated by MSNs + P. **(E)** Roots treated by MSN + SA + G + P produced restricted lesion and recovered. W–P, water treated/mock inoculation; W + P, water treated/*P. cinnamomi* inoculated; SA + P, SA treated/*P. cinnamomi* inoculated; MSNs + P, bare MSN treated/*P. cinnamomi* inoculated; MSN + SA + G + P, MSN + SA + G treated/*P. cinnamomi* inoculated. Images are representative of three biological replicates, each consisting of 10 individual plantlets. Scale bars = 0.5 cm.

As shown in **Figure 4**, roots in all inoculated treatments responded to the pathogen infection and produced lesions. At 6 dpi, the inoculated control (W + P) possessed the most severe symptoms with water-soaked, brown, spreading, long lesions commencing at the root tip, with few lateral roots. Roots in the SA + P treatment exhibited similar symptoms, but with shorter lesions and longer lateral roots compared with the inoculated control. However, MSNs + P and MSN + SA + G + P treatments showed restricted lesions and lateral roots were stimulated. In addition, for the MSN + SA + G + P treatment, the roots recovered and continued to grow.

The average root growth at 2 dpi in all pineapple plants is shown in **Supplementary Figure S4**. Compared to the other treatments, the MSNs–P and MSN + SA + G–P treatments had significantly enhanced root growth over 6 days. However, W + P significantly inhibited the root growth compared with noninoculated control (W–P). At 4 and 6 dpi, the root growth of all the pathogen-inoculated plants was impeded, compared with the controls.

**Figure 5:**
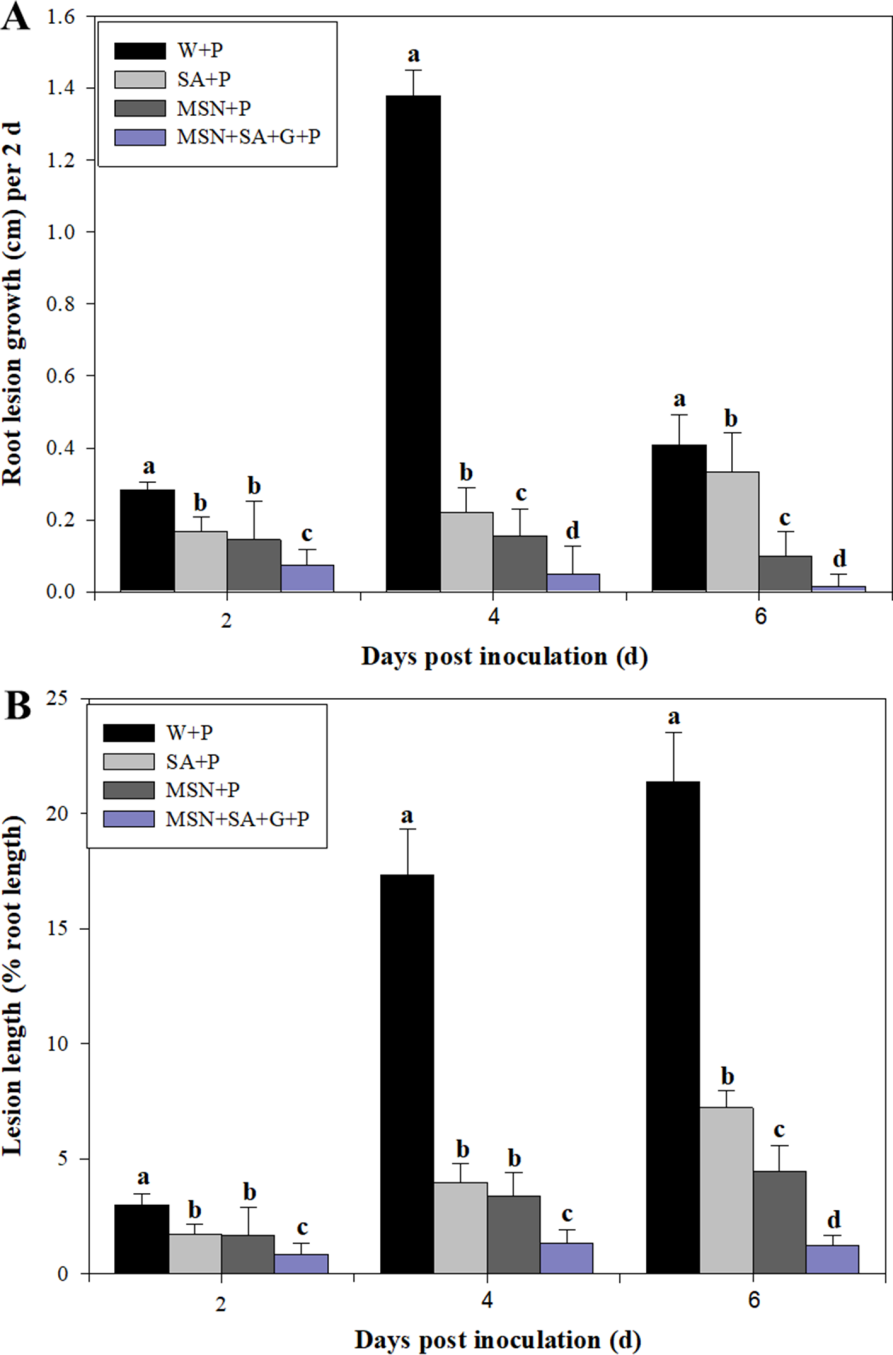
The effect of SA, MSNs, and MSN + SA + G pretreatments on root lesion growth **(A)** and the percentage of lesion length to the root length **(B)** in pineapple root over 6 days after inoculation with *P. cinnamomi*. W + P, water treated/*P. cinnamomi* inoculated; SA + P, SA treated/*P. cinnamomi* inoculated; MSNs + P, bare MSN treated/*P. cinnamomi* inoculated; MSN + SA + G + P, MSN + SA + G treated/*P. cinnamomi* inoculated. Bars with different letters indicate values significantly (*p* < 0.05) different from the control at a given time point, according to Duncan's multiple-range test. Data represent the mean ± SD of three biological replicates, each consisting of 10 individual plantlets.

The lesion lengths caused by *P. cinnamomi* in pineapple roots were recorded every 2 dpi until 6 dpi (**Figure 5A**). The results showed that SA, MSNs, and MSN + SA + G significantly reduced lesion length at all time points. In addition, the MSN + SA + G + P treatment showed a significant reduction in lesion length compared with the other treatments over time. Compared to the W + P treatment, the percentage of lesion length to root length also significantly decreased in the plants treated by SA, MSNs, and MSN + SA + G at varying time points (**Figure 5B**). Moreover, lesions in MSNs and MSN + SA + G–treated plants were observed as restricted infection, and in some MSN + SA + G–treated plants, the infection sites were difficult to observe.

### SA and GSH Quantification After Pathogen Inoculation

**Figure 6:**
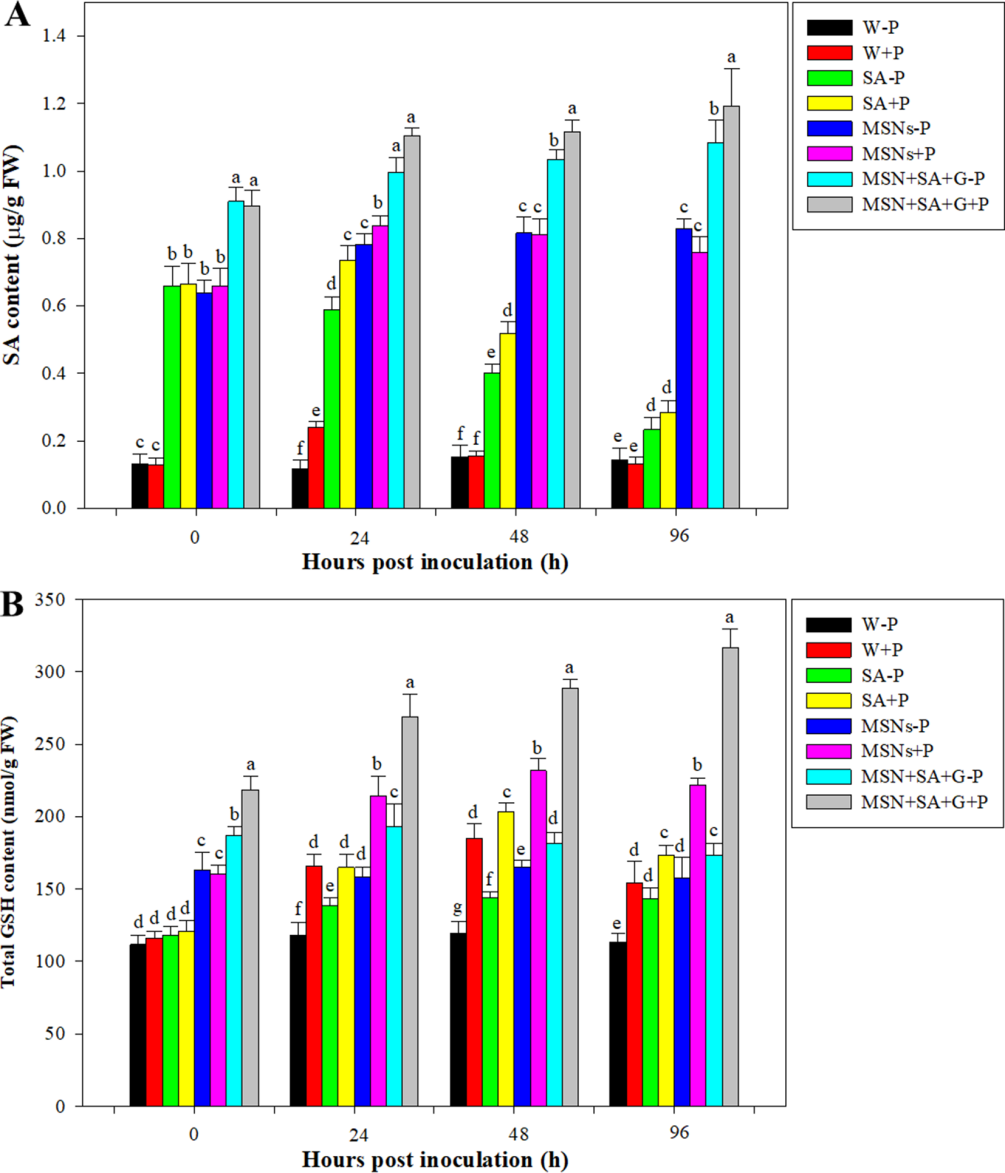
Accumulation of SA **(A)** and GSH **(B)** contents in pineapple roots over 96 hpi pretreated with SA, MSNs, and MSN + SA + G. After 3 days, the plantlets were inoculated with *P. cinnamomi*. W–P, water treated/mock inoculation; W + P, water treated/*P. cinnamomi* inoculated; SA–P, SA treated/mock inoculation; SA + P, SA treated/*P. cinnamomi* inoculated; MSNs–P, bare MSN treated/mock inoculation; MSNs + P, bare MSN treated/*P. cinnamomi* inoculated; MSN + SA + G–P, MSN + SA + G treated/mock inoculation; MSN + SA + G + P, MSN + SA + G treated/*P. cinnamomi* inoculated. Bars with different letters indicate values significantly (*p* < 0.05) different from the control at a given time point, according to Duncan's multiple-range test. Data represent the mean ± SD of three biological replicates, each consisting of 10 individual plantlets.

As shown in **Figure 6A**, the content of SA in control roots remained at a low level for the duration of the experiment. But after pathogen inoculation, the SA level in the W + P treatment significantly increased at 24 hpi and then dropped to the original level. By 24 hpi, the SA content in the SA–P treatment significantly decreased, while in the SA + P, MSNs–P, MSNs + P, MSN + SA + G + P, and MSN + SA + G–P treatments SA continued to increase. Moreover, it was found that the contents of SA in MSN + SA + G–pretreated roots were elevated over the time, and the MSN + SA + G + P treatment significantly improved the SA level in the roots compared with the MSN + SA + G–P treatment at 48 and 96 hpi.

It was found that, by 0 hpi, the GSH levels were increased 1.5-fold under MSNs–P, 1.4-fold under MSNs + P, 1.7-fold under MSN + SA + G–P, and 2.0-fold under MSN + SA + G + P treatment compared with W–P (**Figure 6B**). In addition, a considerable increase from 0 to 24 hpi was observed in the plants treated with MSNs + P, and then the level slightly declined at 48 hpi, remaining the highest GSH content compared to all the other treatments except for the treatment group of MSN + SA + G + P. In the roots exposed to the treatment of MSN + SA + G + P, a continuous increase in GSH content was observed throughout the experiment, and a significantly higher level was found at each the time point compared to that in all the other treatments.

### Resistance-Related Gene Expression Analysis in Pineapple Roots Inoculated With *P. cinnamomi*

**Figure 7:**
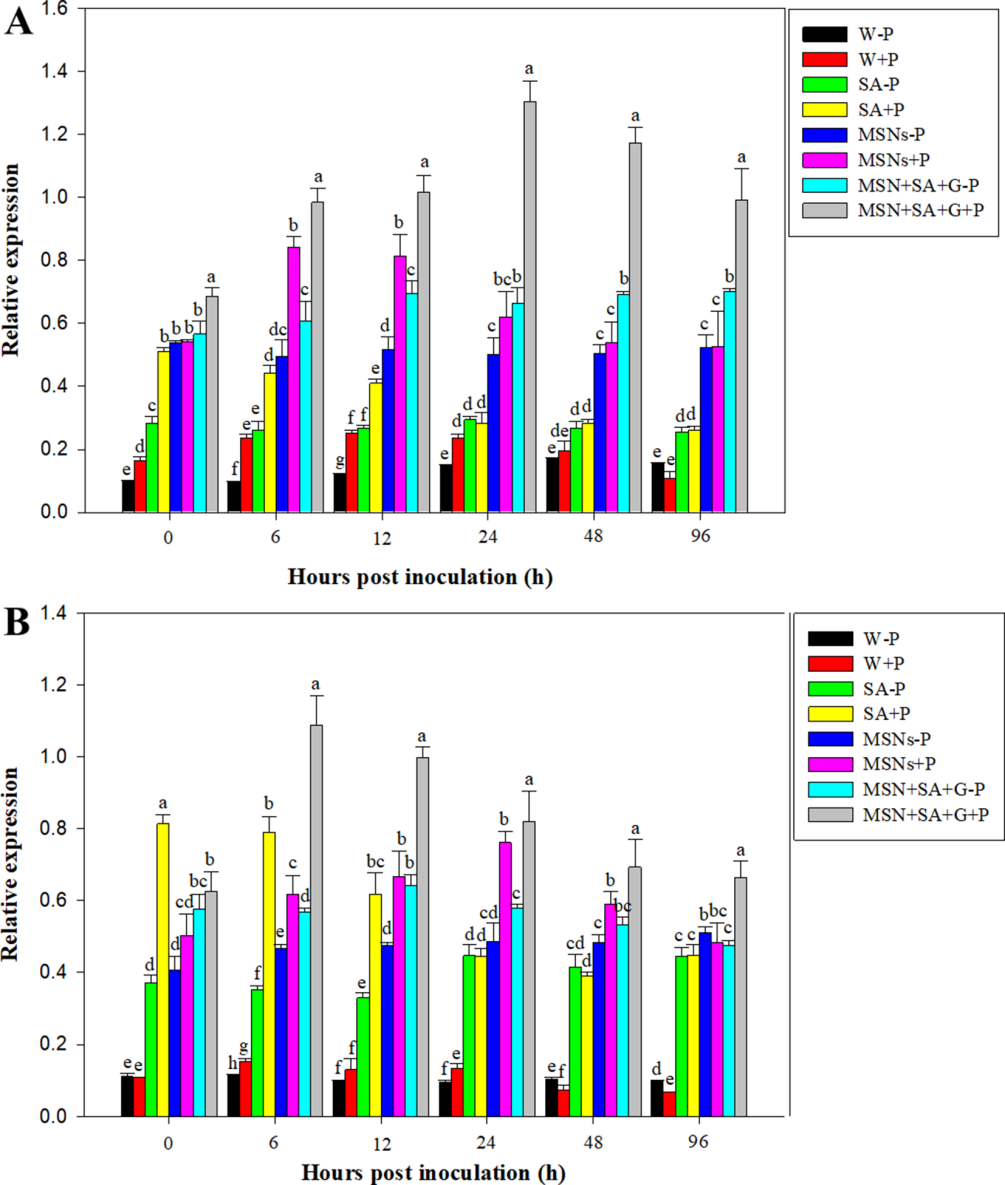
Relative expression of *PR1* **(A)** and *PR5* **(B)** in pineapple roots over 96 hpi pretreated with SA, MSNs, and MSN + SA + G, followed by inoculation with *P. cinnamomi*. Relative expression was measured against *actin* of each individual sample. W–P, water treated/mock inoculation; W + P, water treated/*P. cinnamomi* inoculated; SA–P, SA treated/mock inoculation; SA + P, SA treated/*P. cinnamomi* inoculated; MSNs–P, bare MSN treated/mock inoculation; MSNs + P, bare MSN treated/*P. cinnamomi* inoculated; MSN + SA + G–P, MSN + SA + G treated/mock inoculation; MSN + SA + G + P, MSN + SA + G treated/*P. cinnamomi* inoculated. Bars with different letters indicate values significantly (*p* < 0.05) different from the control at a given time point according to Duncan's multiple-range test. Data represent the mean ± SD of three biological replicates, each consisting of 10 individual plantlets.

As shown in **Figure 7A**, expression of *PR1* in the tissue treated with W + P increased at 12 and 24 hpi and then gradually declined to the same level in W–P treatment at 96 hpi. Salicylic acid + P significantly elevated *PR1* expression at 0 hpi, and about 5.0, 3.5, and 2.0 times higher expression were observed compared to the treatments of W–P, W + P, and SA–P, respectively. After that, *PR1* demonstrated a continuous decrease in expression until 96 hpi. The treatment of MSNs–P increased the expression of *PR1*, and the highest level was detected at 0 hpi. Later on, *PR1* expression remained at a high level with slight variation for all the subsequent time points. In the roots exposed to the treatment of MSNs + P, the highest level of gene expression was achieved at 6 hpi, and then the level gradually declined. A considerably elevated *PR1* expression was observed in the roots treated with MSN + SA + G + P, and the expression levels were obviously higher than that in all the other treatments at all time points.

The overall pattern of *PR5* expression was similar to that of *PR1* expression. *PR5* expression levels were greatly increased in the roots exposed to the treatment of MSN + SA + G + P to levels above that in all the other treatments at all time points after 0h. The highest value was observed at 6 hpi, and then *PR5* expression slowly decreased but was still maintained above the control level until 96 hpi (**Figure 7B**).

## Discussion

It is well recognized that treatment of plants with various biotic and abiotic agents can result in the induction of both local and systematic resistance to subsequent pathogen attack (Palmer et al., 2017). In the present study, in comparison to the other resistance inducers screened, SA at 0.5 to 5.0 mM was found to be more effective in limiting lesion development in lupin caused by *P. cinnamomi*, reduced disease severity, and promoted root growth. A previous study showed that the exposure of avocado roots to SA, for example, induced the accumulation of compounds, which effectively induced resistance to *P. cinnamomi* (Rangel-Sánchez et al., 2014). As a result, SA was taken further to analyze the potential of MSNs as a vehicle for delivering a resistance inducing agent to pineapple, the main aim of this study.

Mesoporous silica nanoparticles with specifically localized functionalization are essential for maintaining the particle size and pore volume. The results from BET/BJH analysis showed that the surface of MSN + SH significantly decreased because of their occupation by thiol groups. The pore volume and pore diameter of MSN + SH did not reduce dramatically, providing effective LC for hosting a guest molecule. Previous research has demonstrated that the size of MSNs could impact on their uptake into plants and further affect the efficacy of the delivery system (Sun et al., 2014). After being loaded with SA, the pore volume of MSNs decreased dramatically, which meant the pores were occupied. The absorption peak between 500 and 550 cm^−1^ in the spectrum of the didecyl disulfide revealed the covalent disulfide bonds, which were generated after oxidation of decanethiol (Gosselin et al., 2007).

High LE of resistance inducers into MSNs is critical for further applications in plants. Studies have indicated that LE was directly related to the mesoporous structure, pore size, surface area, and surface modification of MSNs (Cao et al., 2016). The results presented in the current study showed that the LE of SA was nearly 1.5 times higher than that of the bare MSNs. The improved LC was due to the capping of the pores by the gatekeeper molecules, which blocked the loss of SA and limited premature leakage. The *in vitro* redox-responsive release test demonstrated that the release of SA from decanethiol gatekeeper-covered MSNs slowed down considerably compared to that of MSNs without gatekeepers. Further, the release rate of the entrapped SA improved with the increase in the levels of GSH stimulus.

Resistance inducer carriers with controllable release in response to environmental stimuli are extremely critical for improving the efficiency of loaded molecules. In this study, the disulfide-linked gatekeeper-capped MSNs comprise the mesopores of MSNs as a reservoir for resistance inducers, the disulfide bond as a redox-responsive cleavable linker, and the decanethiol “gatekeeper” that can cover the entrance of MSNs. Here, the concentrations of GSH played a critical role in determining the SA release rate. The higher concentrations of GSH quickly cleaved the disulfide bonds between decanethiol and MSNs, opening the space for the release of SA. Thus, the gatekeeper-capped MSNs could be a potential delivery system in that resistance inducers can be encapsulated without being prematurely released until the gatekeeper is removed by GSH, which accumulates to high concentrations in plants under stress conditions (Agarwal, 2007; Arasimowicz et al., 2014; Zechmann, 2014).

More interestingly, MSN treatments resulted in significant improvement in root growth. These beneficial effects are mainly attributed to silica, the major component of silica nanoparticles (SNPs), which play important roles in enhancing plant growth and improving plant resistance (Yuvakkumar et al., 2011; Li et al., 2012; Suriyaprabha et al., 2014a; Sun et al., 2016). In addition, MSNs alone evidently reduced lesion development. Previous studies have shown that SNPs could increase the resistance of plants to various biotic and abiotic stresses such as salinity in bean (*Phaseolus vulgaris*) (Alsaeedi et al., 2017), UV-B stress in wheat (*Triticum aestivum*) seedlings (Tripathi et al., 2017), and fungal attack in maize (Suriyaprabha et al., 2014b), which might have protected plants *via* triggering the plant defense system, enhancing antioxidants and phenolic compounds, and alleviating reactive oxygen species-induced damage to photosystems. Moreover, MSNs treatment induced the accumulation of SA in the treated roots. This might be attributed to the silica elements from MSNs, which could stimulate the biosynthesis of the stress hormones such as SA, JA, and ET in the treated plants (Fauteux et al., 2006).

Studies have shown that the exogenous application of SA can trigger SAR against a wide range of pathogens that cause disease in many plant species (Faize and Faize, 2018). In the present study, a constant increase and high level of SA in the tissue exposed to the treatments with the application of the MSN-mediated delivery system were observed. So, a sustained release of entrapped SA from the mesopores was achieved, thus improving the plant’s overall resistance. It is recognized that during interactions with pathogens, GSH is not only involved in signaling roles but also related to hormone and secondary metabolite synthesis. In general, a certain level of GSH is needed for the synthesis of pathogen defense-related molecules and disease resistance (Ghanta and Chattopadhyay, 2011). In the present study, in the roots exposed to SA encapsulated in MSNs, the GSH level was higher than all the other treatment groups during all experiments and continuously increased until the end of the experiment. The exogenous use of SA may also enhance GSH levels in treated plants, indicating a physiological interaction between GSH and SA levels (Freeman et al., 2005; Mateo et al., 2006). Hence, the changes in GSH levels in the treated roots further confirmed the success of this MSN-mediated delivery system for the controlled release of SA.

The synthesis of pathogenesis-related proteins PR1 and PR5 are an example of inducible defense mechanisms that plants can trigger to resist pathogen invasion, and their expression has been directly correlated with the developmental transition to resistance (Ponciano et al., 2006; Engelbrecht and van den Berg, 2013). In the current study, the application of SA and MSNs elevated the transcription of *PR1* and *PR5* both in infected and noninfected roots. In addition, a similar result was observed in the expression of *PR5* except that free SA introduced a significantly higher expression at the beginning, and the peak of *PR5* expression occurred at 12 h. The sustained and elevated expression of *PR1* and *PR5* was attributed to a constant supply of SA released from the pores of the MSNs. Similar to these results, a recent study showed the expression of the ABA-induced gene *AtGALK2* in *Arabidopsis* was prolonged due to the application of an MSN-mediated ABA delivery system (Sun et al., 2018). Together, in the present study, *PR1* and *PR5* gene expression levels, and SA and GSH quantification demonstrated that the MSN-mediated delivery system provided a sustained release of SA to plants, offering prolonged protection against pathogen attack.

## Conclusion

The MSN-mediated delivery system with short-chain molecules gated as redox-responsive gatekeepers was synthesized and applied for the control of plant disease. The *in vitro* release experiments indicated that decanethiol gatekeepers effectively blocked the loaded SA inside MSNs without the presence of GSH. However, the disulfide bonds between decanethiol and MSNs were cleaved with the presence of GSH, resulting in the release of entrapped SA. Further, *in planta* experiments demonstrated that the controlled release of SA from decanethiol-gated MSNs induced sustained expression of the plant defense genes *PR1* and *PR5* and improved pineapple plantlet resistance to root rot disease caused by *P. cinnamomi*. Hence, in the present study, the application of MSNs with redox-responsive gatekeepers has been demonstrated to be an efficient technique to introduce resistance inducers into plants and release them in a controllable fashion.

## Data Availability Statement

All datasets generated for this study are included in the manuscript and the **Supplementary Files**.

## Author Contributions

XL, LK, and DC proposed and designed the experiments. XL carried out experiments. XL and DS analyzed data and wrote the paper. JR and DC revised the manuscript. XZ helped in finalizing the manuscript and secured funding. All authors read and approved the final manuscript.

## Conflict of Interest

The authors declare that the research was conducted in the absence of any commercial or financial relationships that could be construed as a potential conflict of interest.
