# Supplementary figures and images for: Nanoapplication of a Resistance Inducer to Reduce Phytophthora Disease in Pineapple (Ananas comosus L.)
Source: Front Plant Sci. 2019 Oct 11;10:1238. doi: 10.3389/fpls.2019.01238 (PMC6797602; doi:10.3389/fpls.2019.01238)

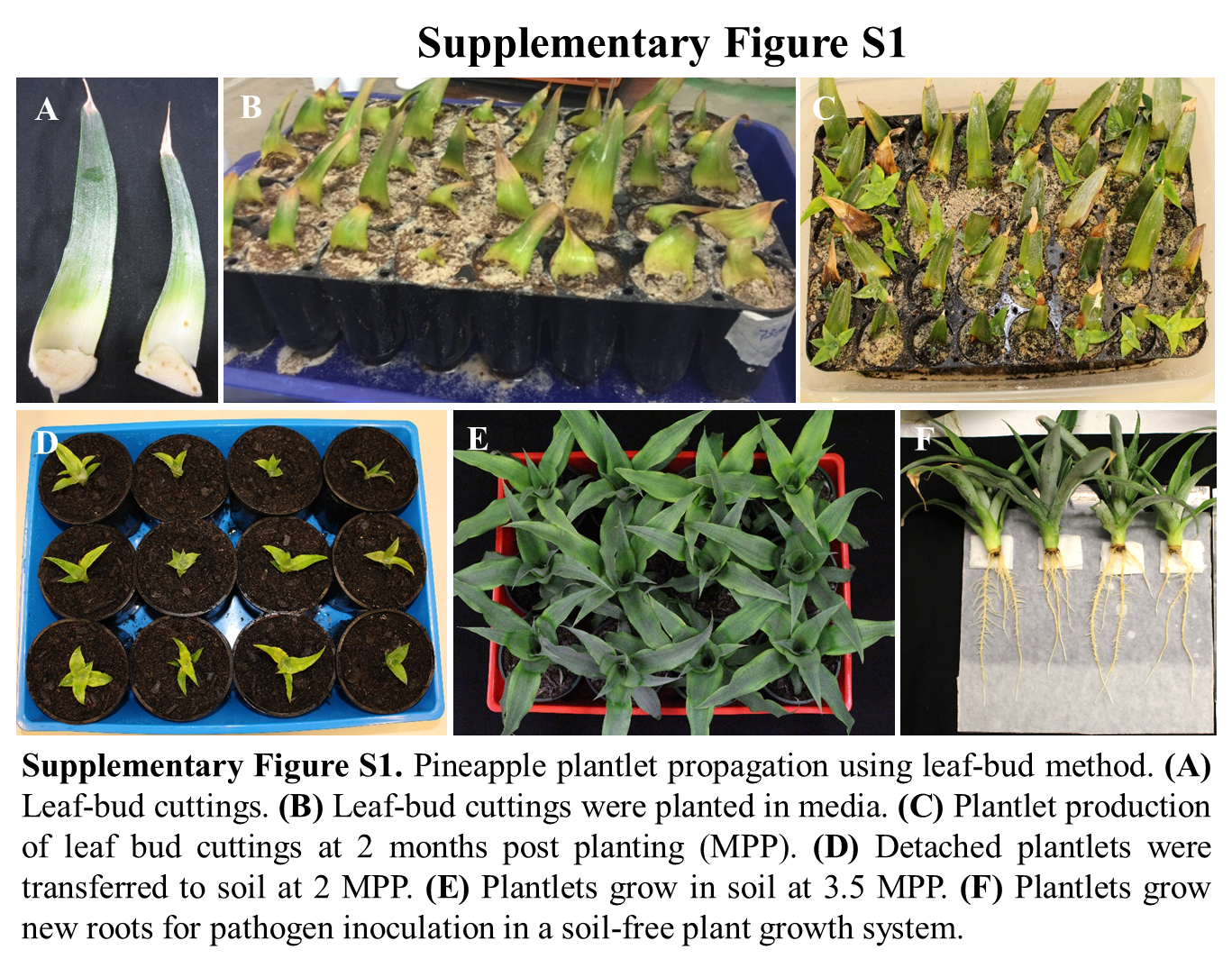

Supplement: Supplementary file 1 [file Image_1.tif]

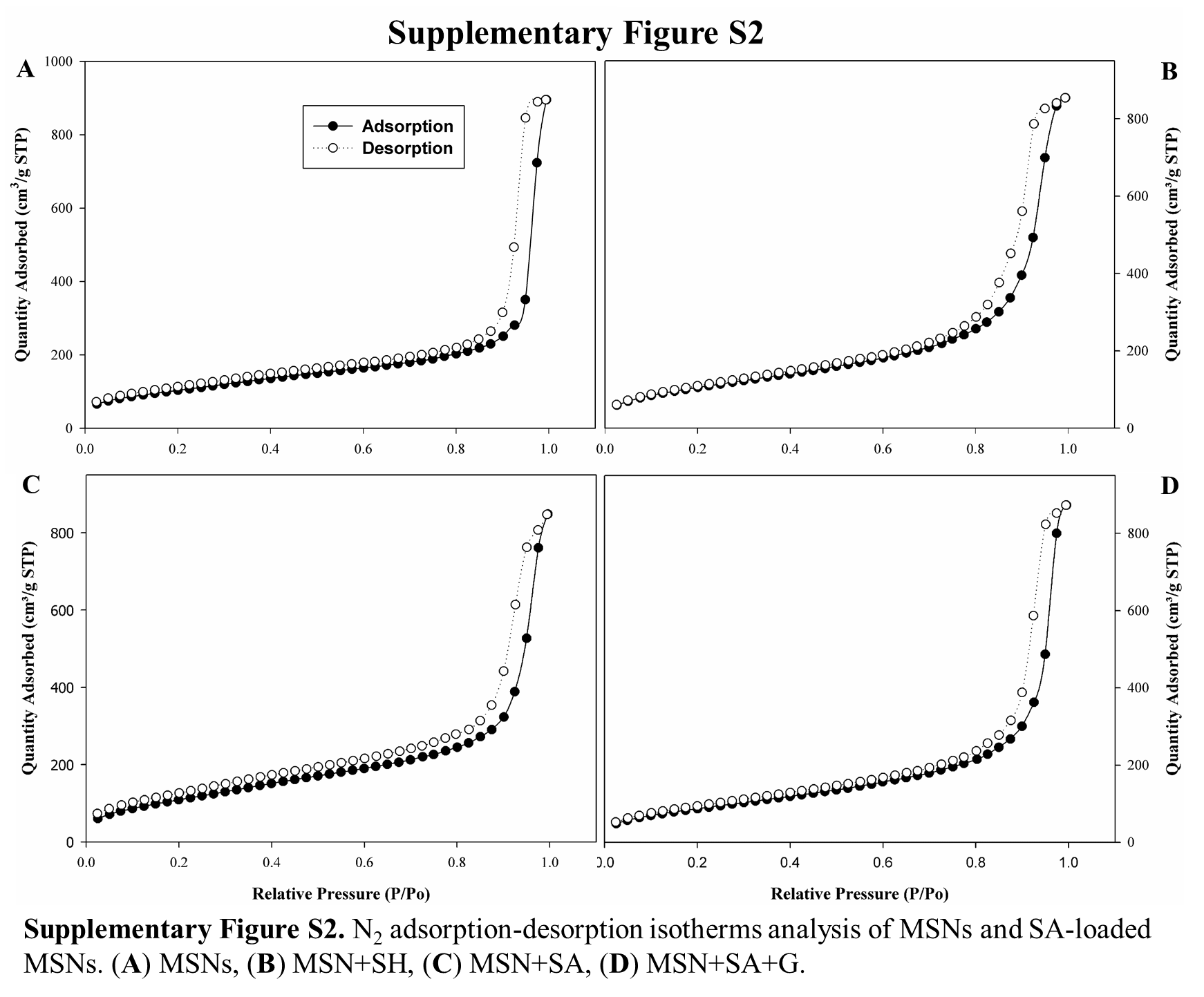

Supplement: Supplementary file 2 [file Image_2.tif]

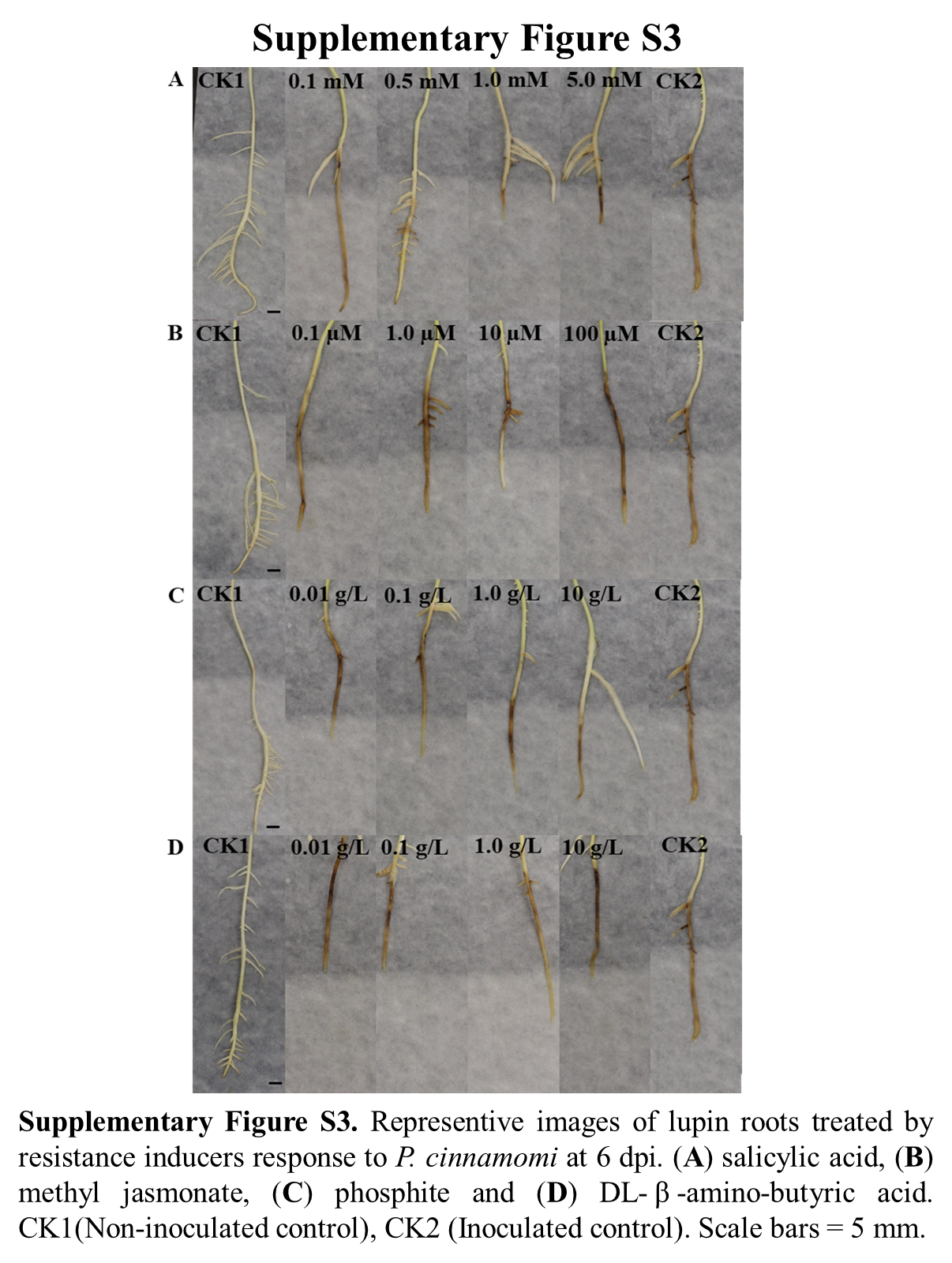

Supplement: Supplementary file 3 [file Image_3.tif]

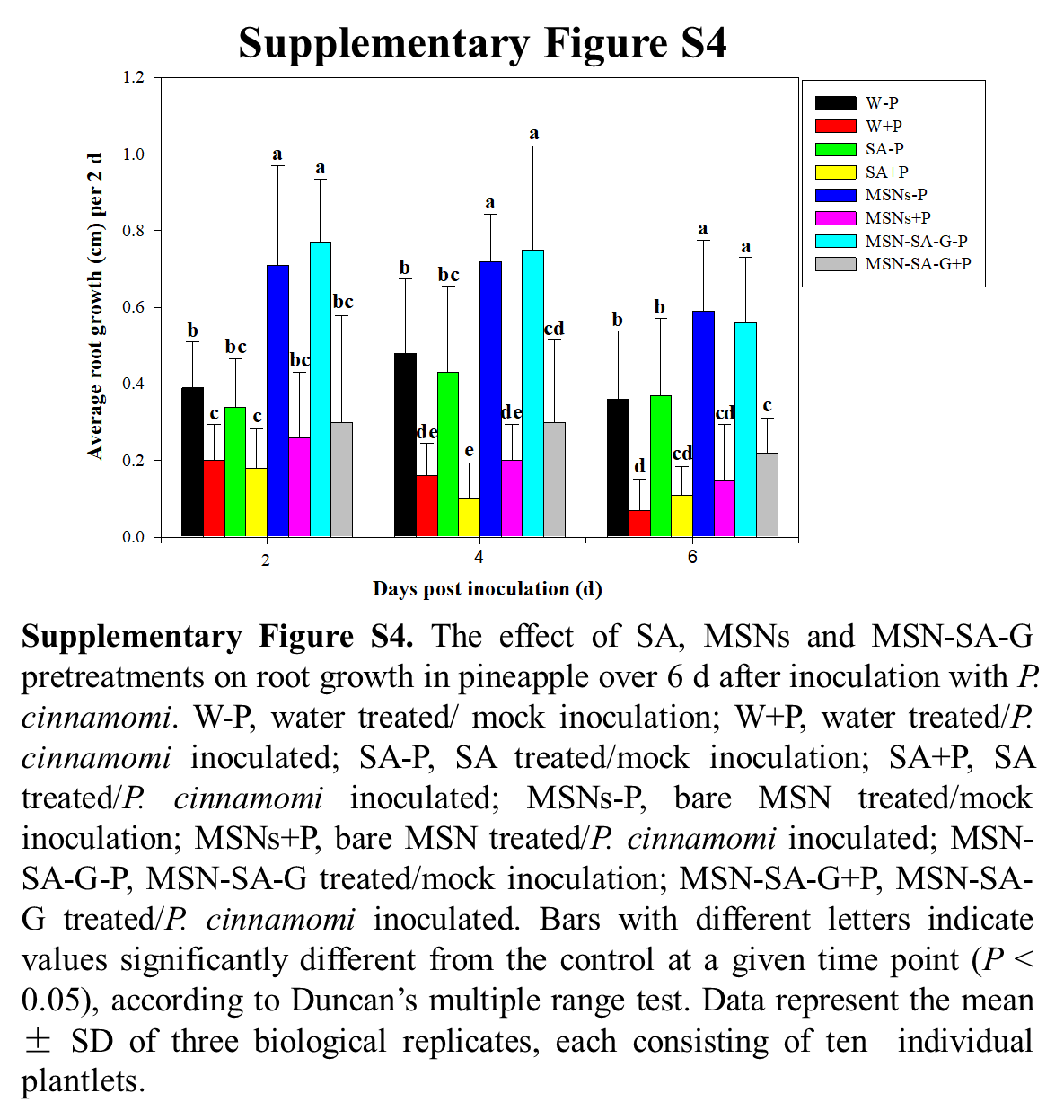

Supplement: Supplementary file 4 [file Image_4.tif]
