# Supplementary material for: Nanoapplication of a Resistance Inducer to Reduce Phytophthora Disease in Pineapple (Ananas comosus L.)
Source: Front Plant Sci. 2019 Oct 11;10:1238. doi: 10.3389/fpls.2019.01238 (PMC6797602; doi:10.3389/fpls.2019.01238)
Supplement: Supplementary file 5 [file Table_1.docx]

**Table S1**. Surface area, pore volume and pore diameter of different particle types.

Samples Surface area (m^2^/g) Pore volume (cm^3^/g) Pore diameter (nm)

MSNs 616.2 ± 0.9 0.369 ± 0.008 3.0 ± 0.2

MSN+SH 397.4 ± 4.2 0.257 ± 0.005 2.7 ± 0.3

MSN+SA 302.4 ± 6.5 0.121 ± 0.001 2.2 ± 0.1

MSN+SA+G 333.8 ± 2.5 0.057 ± 0.001 -

Note: Data represent the mean ± SD of three biological replicates.
