# Supplementary material for: Nanoapplication of a Resistance Inducer to Reduce Phytophthora Disease in Pineapple (Ananas comosus L.)
Source: Front Plant Sci. 2019 Oct 11;10:1238. doi: 10.3389/fpls.2019.01238 (PMC6797602; doi:10.3389/fpls.2019.01238)
Supplement: Supplementary file 6 [file Table_2.docx]

**Table S2.** Effect of treatments with resistance inducers on lupin roots inoculated by *P. cinnamomi* at 6 dpi.

**A**

SA Disease severity grade Root length Lesion length LL/RL Lateral root formation**

(mM) and symptom* (cm) (cm) (%)

CK1 0 17.8 ± 0.9 - - High

CK2 5, spreading 12.2 ± 0.9bc 6.7 ± 0.5a 55.2 ± 4.7a Medium

0.1 4, spreading 13.3 ± 1.2ab 6.4 ± 0.4a 48.1 ± 1.8b Low

0.5 1, spot infection^***^ 14.5 ± 1.3a 1.3 ± 0.1c 8.7 ± 0.5d High

1.0 1, restricted 13.1 ± 2.0abc 1.8 ± 0.2c 13.6 ± 1.3c Medium

5.0 4, restricted 11.3 ± 1.7c 5.2 ± 0.6b 46.1 ± 2.3b Medium

**B**

MeJA Disease severity grade Root length Lesion length LL/RL Lateral root formation

(µM) and symptom (cm) (cm) (%)

CK2 5, spreading 12.2 ± 0.9 6.7 ± 0.5a 55.2 ± 4.7a Medium

0.1 4, spreading 12.6 ± 1.4 5.5 ± 0.8bc 43.3 ± 4.0cd Low

1.0 4, spreading 12.2 ± 1.3 5.8 ± 0.6b 47.6 ± 2.8bc Medium

10.0 3, restricted 12.4 ± 2.5 4.8 ± 0.6c 39.1 ± 0.8d Medium

100.0 4, spreading 12.0 ± 0.9 6.1 ± 0.9ab 50.6 ± 5.1ab No

**C**

Phi Disease severity grade Root length Lesion length LL/RL Lateral root formation

(g/L) and symptom (cm) (cm) (%)

CK2 5, spreading 12.2 ± 0.9 6.7 ± 0.5a 55.2 ± 4.7a Medium

0.01 5, spreading 11.8 ± 1.2 6.4 ± 0.5a 54.2 ± 3.6a Low

0.1 5, spreading 12.4 ± 1.2 6.5 ± 0.5a 52.9 ± 4.1a Medium

1.0 4, spreading 12.0 ± 1.0 5.7 ± 0.6b 47.9 ± 1.3b Medium

10.0 3, spreading 12.1 ± 1.1 3.8 ± 0.2c 31.2 ± 1.4c Low

**D**

BABA Disease severity grade Root length Lesion length LL/RL Lateral root formation

(g/L) and symptom (cm) (cm) (%)

CK2 5, spreading 12.2 ± 0.9 6.7 ± 0.5a 55.2 ± 4.7a Medium

0.01 5, spreading 12.1 ± 0.2 6.2 ± 0.7ab 51.6 ± 5.2a Low

0.1 4, spreading 11.7 ± 1.1 5.4 ± 0.5cd 46.3 ± 2.6bc High

1.0 4, spreading 11.1 ± 0.6 4.8 ± 0.3d 43.0 ± 2.5c Medium

10.0 5, spreading 11.4 ± 1.0 5.7 ± 0.5bc 50.2 ± 3.4ab Low

**Note:** CK1(Non-inoculated control), CK2 (Inoculated control), LL/RL (Lesion length/Root length). Data represent the mean ± SD of three biological replicates, each consisting of twelve individual plantlets. Different letters in each column indicate statistical differences (*P* <0.05, according to Duncan's multiple range test).

*Disease severity was graded from 0 to 5 according to lesion length: 0, no visible symptoms; 1, mild, lesion length on ˂20% of the root length; 2, mild to moderate, lesion length on 20-30 % of the root length; 3, moderate, lesion length on 30-40% of the root length; 4, moderate to severe, lesion length on 40-50% of the root length; 5, severe, lesion length on >50% of the root length.

** Numbers of lateral roots formed at 6dpi. Low, numbers of lateral roots ˂5; medium, numbers of lateral roots 5-10; high, numbers of lateral roots >10.

***Lesion is restricted to two sides of the inoculation point.
