# Supplementary material for: Nanoapplication of a Resistance Inducer to Reduce Phytophthora Disease in Pineapple (Ananas comosus L.)
Source: Front Plant Sci. 2019 Oct 11;10:1238. doi: 10.3389/fpls.2019.01238 (PMC6797602; doi:10.3389/fpls.2019.01238)
Supplement: Supplementary file 7 [file Table_3.docx]

**Table S3**. The SA loading efficiency and capacity in NPs

Samples Loading efficiency (%) Loading capacity (µg SA/mg NPs)

MSN+SA 8.0 ± 0.7b 88.8 ± 2.5b

MSN+SA+G 11.7 ± 0.5a 134.1 ± 12.7a

Note: Data represent the mean ± SD of three biological replicates. Different letters in each column indicate statistical differences (P <0.05, according to Duncan's multiple range test).
